# Key Strategies on Cu_2_O Photocathodes toward Practical Photoelectrochemical Water Splitting

**DOI:** 10.3390/nano13243142

**Published:** 2023-12-15

**Authors:** Min-Kyu Son

**Affiliations:** Nano Convergence Materials Center, Emerging Materials R&D Division, Korea Institute of Ceramic Engineering & Technology (KICET), Jinju 52851, Republic of Korea; minkyu.son@kicet.re.kr; Tel.: +82-55-792-2683

**Keywords:** cuprous oxide, photocathode, photoelectrochemical water splitting, back contact layer, overlayer, protection layer, hydrogen evolution reaction catalyst

## Abstract

Cuprous oxide (Cu_2_O) has been intensively in the limelight as a promising photocathode material for photoelectrochemical (PEC) water splitting. The state-of-the-art Cu_2_O photocathode consists of a back contact layer for transporting the holes, an overlayer for accelerating charge separation, a protection layer for prohibiting the photocorrosion, and a hydrogen evolution reaction (HER) catalyst for reducing the overpotential of HER, as well as a Cu_2_O layer for absorbing sunlight. In this review, the fundamentals and recent research progress on these components of efficient and durable Cu_2_O photocathodes are analyzed in detail. Furthermore, key strategies on the development of Cu_2_O photocathodes for the practical PEC water-splitting system are suggested. It provides the specific guidelines on the future research direction for the practical application of a PEC water-splitting system based on Cu_2_O photocathodes.

## 1. Introduction

### 1.1. Basic Principles of Photoelectrochemical Water Splitting

**Figure 1:**
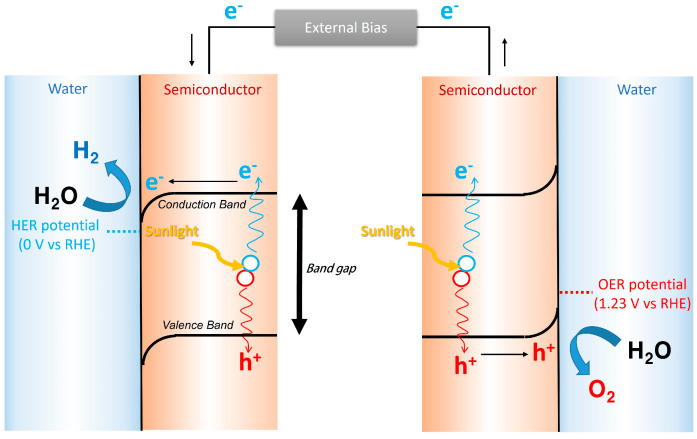
Operational principle of PEC water splitting with two semiconductor electrodes.

Photoelectrochemical (PEC) water splitting has been regarded as an ideal technology for generating CO_2_-free hydrogen because it does not emit CO_2_ as a by-product during PEC operation, contrary to traditional hydrogen-production technologies based on fossil fuel. Figure 1 illustrates an operational principle of the PEC water-splitting system with two semiconductor electrodes. The overall PEC water-splitting reaction includes three steps [1,2,3,4]: (1) Electron-hole pairs are generated when sunlight is absorbed by the semiconductor in the water. (2) After separating the generated electron-hole pairs by the internal electric field in the depletion layer or the external bias, electrons in the conduction band (CB) of the semiconductor and holes in the valence band (VB) of the semiconductor drift to the semiconductor/water interface. (3) Water splits into hydrogen and oxygen by electrons and holes, respectively, at the semiconductor/water interfaces via the following reactions.

2H^+^ + 2e^−^ → H_2_ (Hydrogen evolution reaction, HER) (1)

2H_2_O + 4h^+^ → 4H^+^ + O_2_ (Oxygen evolution reaction, OER) (2)

2H_2_O → 2H_2_ + O_2_ (Overall water-splitting reaction) (3)

Semiconductors are a key component in the PEC water-splitting system because it absorbs sunlight, and moreover water-splitting reactions occurs at its interface. There are two criteria of a semiconductor for overall PEC water splitting according to the operational principle of PEC water splitting, as illustrated in Figure 1 [1,5,6,7,8]. First, its CB energy level (E_CB_) should be lower than the HER potential (0 versus reversible hydrogen electrode, RHE), while its VB energy level (E_VB_) should be higher than the OER potential (1.23 V versus RHE).

E_CB_ < 0 V versus RHE, E_VB_ > 1.23 V versus RHE (4)

Second, its band gap (E_g_) should theoretically be larger than 1.23 eV because the difference between the HER potential and OER potential is 1.23 eV. Practically, an Eg of 1.5~1.8 eV is necessary due to the HER/OER overpotentials (η_HER_ and η_OER_) [9,10].

E_g_ > 1.23 eV + η_HER_ + η_OER_ (5)

**Figure 2:**
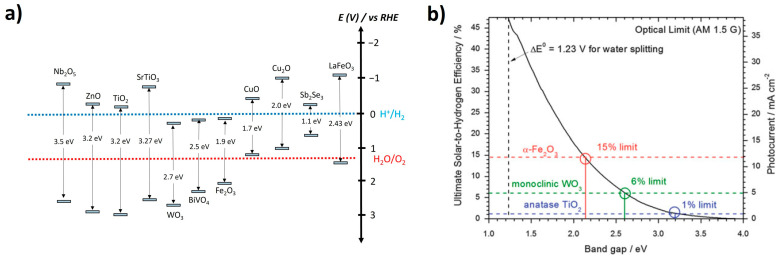
(**a**) Band-gap information of typical PEC semiconductors. It is drawn based on the information in the reported literature [11,12,13,14,16,17,18]. (**b**) Estimated STH efficiency and photocurrent from the band gap of semiconductors. Reprinted from [15] with permission from Springer Nature, copyright 2013.

Among various semiconductors, few wide-band-gap semiconductors, such as TiO_2_ [11], ZnO [12], SrTiO_3_ [13], and Nb_2_O_5_ [14], meet these criteria, as shown in Figure 2a. Photoelectrodes with these materials can complete the entire PEC water-splitting reactions using only sunlight without external bias. However, they can absorb only ultraviolet light below 400 nm due to their band gaps above 3.0 eV, thereby utilizing the limited solar energy. Hence, PEC photoelectrodes based on these wide-band-gap semiconductors show a low solar-to-hydrogen (STH) efficiency below 3% (Figure 2b) [15]. Therefore, semiconductors with a short band gap are necessary to utilize the sufficient solar energy for efficient PEC water splitting.

**Figure 3:**
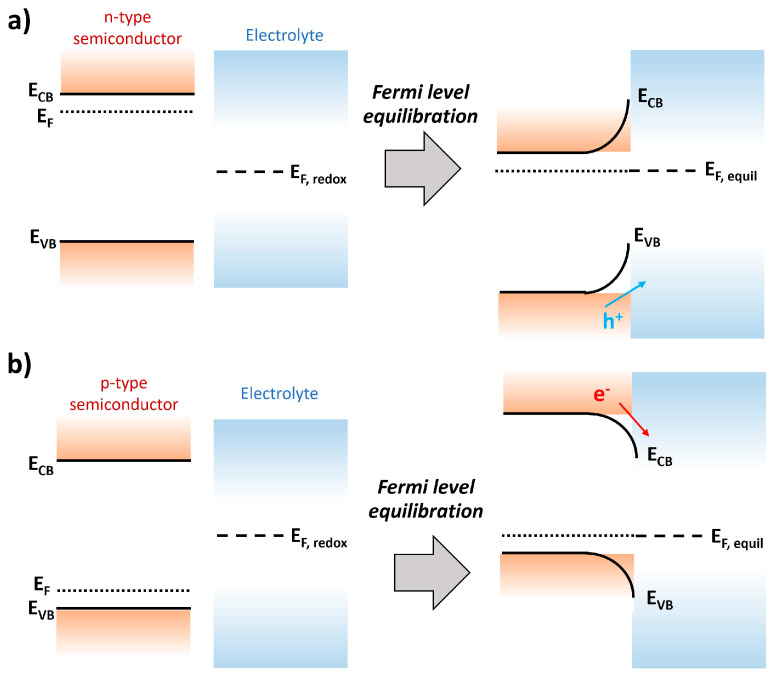
Energy levels of the semiconductor ((**a**) n-type semiconductor and (**b**) p-type semiconductor) and electrolyte before and after Fermi level equilibration (E_F,equil_). E_CB_ is an energy level of the conduction band, E_VB_ is an energy level of the valence band, E_F_ is the Fermi level, and E_F,redox_ is a redox potential of the electrolyte.

Intrinsic characteristics of short-band-gap semiconductors determine the charge transport direction. Figure 3 shows the energy levels of the semiconductor and electrolyte before and after contacting each other, depending on the intrinsic characteristics of the semiconductor. As illustrated in Figure 3, the downward band bending is formed when n-type semiconductors contact the electrolyte, while the upward band bending is formed when p-type semiconductors contact the electrolyte, due to the Fermi level equilibration between the semiconductor and electrolyte. Due to this band bending, holes in the n-type semiconductor easily move to the semiconductor/electrolyte interface, whereas electrons in the p-type semiconductor quickly move to the semiconductor/electrolyte interface. Therefore, in general, n-type semiconductors such as BiVO_4_ [19,20,21], WO_3_ [22,23,24], Fe_2_O_3_ [25,26,27], and Ag_3_PO_4_ [28] have been used as photoanodes for generating oxygen, while p-type semiconductors such as CuO [29,30,31], Cu_2_O [17,32,33,34], Sb_2_Se_3_ [35,36,37], LaFeO_3_ [18,38,39], ternary copper oxide [40], and transitional metal dichalcogenides [41] have been used as photocathodes for generating hydrogen.

### 1.2. Cuprous Oxide Photocathode

**Figure 4:**
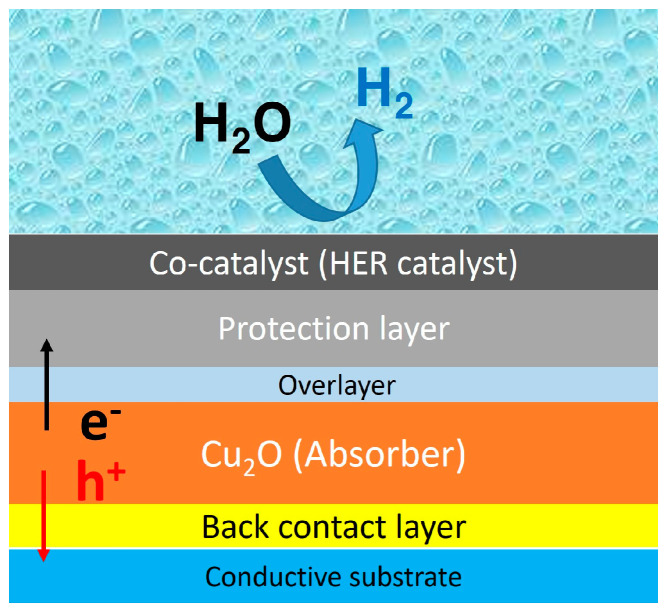
Schematic structure of the state-of-the-art Cu_2_O photocathode.

Cuprous oxide (Cu_2_O) is a remarkable photocathode material for the efficient and economical PEC water-splitting system. Its intrinsic characteristics and energy level are suitable for generating hydrogen via water reduction reactions at the interface. As shown in Figure 2, its E_CB_ is much lower than the HER potential, which is advantageous for transporting electrons to the semiconductor/water interface. In addition, it is possible to utilize the visible light due to its band gap (2 eV). Theoretically, it enables Cu_2_O photocathodes to produce a high photocurrent density up to −14.7 mA cm^−2^, corresponding to a solar-to-hydrogen (STH) efficiency of 18% [15]. Furthermore, it is cheap and earth-abundant. However, it is still challenging to achieve an efficient and durable PEC performance using only a Cu_2_O photoelectrode. Hence, the state-of-the-art Cu_2_O photocathode consists of a back contact layer, overlayer for the heterostructure, protection layer, and HER catalysts, as well as a Cu_2_O light absorber, as illustrated in Figure 4 [32,42,43,44].

In this review paper, the fundamentals of each component in the Cu_2_O photocathode mentioned above are briefly introduced. In addition, recent research progress on these components for the efficient and durable Cu_2_O photocathode is summarized. Further, recent efforts in practical PEC water splitting based on Cu_2_O photocathodes are reviewed. Finally, the future outlook and research directions on the Cu_2_O photocathodes toward practical PEC water splitting are discussed. It will give comprehensive guidelines and insights on the efficient and durable Cu_2_O photocathode to researchers focusing on the practical PEC water-splitting system.

## 2. Fundamentals and Research Progress

### 2.1. Cu_2_O Light Absorber

Cu_2_O is a key component of Cu_2_O photocathodes because it not only generates electron-hole pairs by absorbing sunlight but also transports the generated charges. Therefore, its optical and charge transport characteristics are a significant parameter affecting the PEC performance of Cu_2_O photocathodes. Based on the absorption coefficient, thick film above 1 μm is necessary to absorb sufficient light using the Cu_2_O light absorber [45,46,47]. However, it generally has a limited minority carriers (electrons) diffusion length below 200 nm [45,48,49]. In this regard, the generated electrons in the thick Cu_2_O film are easily recombined before reaching the water interface, irrespective of enough light utilization. On the other hand, the light utilization is not sufficient in the thin Cu_2_O film, even though it is advantageous for efficient electron transport. Therefore, it is necessary to overcome this mismatch for achieving the high PEC performance of Cu_2_O photocathodes.

**Figure 5:**
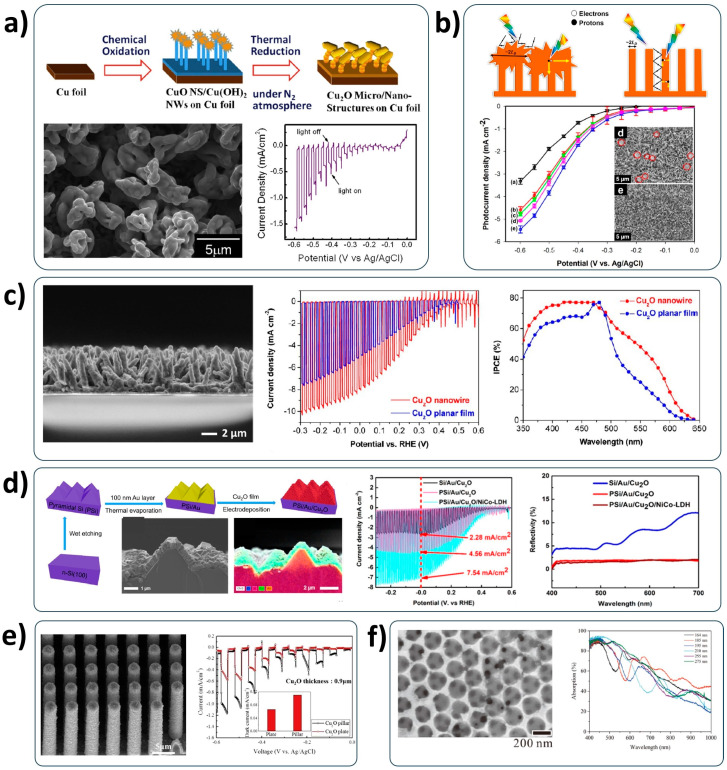
Nanostructure Cu_2_O photocathodes. (**a**) Cu_2_O micro/nanostructured photocathode fabricated by chemical oxidation and thermal reduction. Reprinted from [50] with permission from Elsevier Ltd., copyright 2013. (**b**) Cu_2_O microflowers/nanowires photocathode. Reprinted from [51] with permission from Elsevier Ltd., copyright 2018. (**c**) Cu_2_O nanowire photocathode fabricated by the anodization and annealing process. Reprinted with permission from [52]. Copyright 2016 American Chemical Society. (**d**) Pyramidal Cu_2_O photocathode assisted with the pyramidal silicon template. Reprinted with permission from [53]. Copyright 2022 American Chemical Society. (**e**) Cu_2_O micro pillar photocathode. Reprinted from [54] with permission from Elsevier B.V., copyright 2016. (**f**) Inverse-opal Cu_2_O photocathode assisted with a polystyrene microsphere template. Reprinted from [55] with permission from Elsevier B.V., copyright 2023.

The introduction of nanostructure is an effective strategy to enhance the light utilization by the light-trapping effect. Cu_2_O nanowire structures have been widely adopted in the Cu_2_O photocathode because of the simple fabrication process. In general, the metallic Cu substrate is transformed into the copper hydroxide (Cu(OH)_2_) nanowire electrode by the chemical reactions. Subsequently, it is converted into the Cu_2_O nanowire electrode via the thermal treatment. Hsu et al. fabricated Cu_2_O micro/nanostructured photocathodes by chemical oxidation and subsequent thermal reduction under a N_2_ atmosphere, as shown in Figure 5a [50]. They found that the temperature of thermal reduction is crucial to obtain pure and well-structured Cu_2_O nanowire photocathodes. However, their devices had flower-like Cu_2_O structures with micro scales (2~3 μm) on Cu_2_O nanowires, thereby showing the limited PEC performance, corresponding to an STH efficiency of 1.97%. Salehmin et al. also demonstrated that the coverage of Cu_2_O microflowers on the Cu_2_O nanowires results in the reduced PEC performance because it interrupts the light penetration into the nanowire structure and it prolongs the electron transport length [51]. They fabricated vertically aligned Cu_2_O nanowire photocathodes by controlling the ageing time of nanowire growth. It showed a better PEC performance, resulting from the improved charge transport and prolonged light penetration path, compared to one in the work by Hsu et al. [50] (Figure 5b).

Luo et al. developed high-quality Cu_2_O nanowire photocathodes with excellent electronic and photonic properties by an anodization process in a strong alkaline solution and an annealing process under an Ar atmosphere [52]. The pure Cu_2_O nanowire photocathode was successfully completed using the Cu sputtered fluorine-doped tin oxide (FTO) substrate with a thickness of Cu above 1.5 μm. Such a thick Cu layer continuously provides the Cu source for the transformation of Cu(OH)_2_ into pure Cu_2_O during the annealing process. In addition, they electrodeposited the Cu_2_O thin layer on the Cu_2_O nanowire photocathode as a blocking layer for preventing the charge recombination. As shown in Figure 5c, the improved incident-photon-to-current efficiency (IPCE) in the longwave length region (500~600 nm) was observed in their Cu_2_O nanowire photocathodes, due to the light-trapping effect by the nanowire structure. As a result, it showed a remarkably improved PEC performance compared to the planar Cu_2_O photocathodes. A current density was achieved up to −8 mA cm^−2^ at the HER potential, corresponding to an STH efficiency of 10%, combined with an aluminum-doped zinc oxide (AZO) overlayer and ruthenium oxide (RuO_x_) HER catalysts. Interestingly, the diameter of the Cu_2_O nanowire in this literature is within the minority carrier diffusion length of Cu_2_O. Therefore, the nanowire structure is beneficial for better charge transport, as well as improved light utilization.

Recently, various template-assisted Cu_2_O nanostructure photocathodes have been also introduced. Zhao et al. developed pyramidal Cu_2_O photocathodes assisted with the pyramidal silicon (Si) template [53]. It was fabricated by electrodepositing Cu_2_O film on the gold (Au) layered pyramidal Si template. The pyramidal structure exhibited a significantly reduced reflectance in the longwave length region above 500 nm (Figure 5d). This means that it is effective to enhance the light utilization by the light-trapping effect. Cu_2_O micro pillar photocathodes was successfully fabricated by Yoo’s group [54]. They used the nickel (Ni) micro pillars with heights of 16 μm and diameters of 1~1.5 μm as a template for synthesizing Cu_2_O micro pillar photocathodes. Contrary to other Cu_2_O nanowire photocathodes, it was aligned well perpendicularly to the substrate, as shown in Figure 5e. It provides more active sites for water reduction reactions, which is favorable to improve the PEC performance. Inverse-opal is a typical 3D nanostructure for improving the light utilization in photonic devices. Wu et al. recently developed Cu_2_O inverse-opal photocathodes using polystyrene (PS) microspheres as a colloidal template [55]. The inverse-opal Cu_2_O photocathode was successfully fabricated by removing the PS microsphere template after depositing a Cu_2_O film by the electrochemical deposition method. The light absorption characteristics of the inverse-opal Cu_2_O structure were dependent on the size of the PS microsphere template (Figure 5f). Based on this observation, they successfully optimized the inverse-opal structure for efficient Cu_2_O photocathodes.

**Figure 6:**
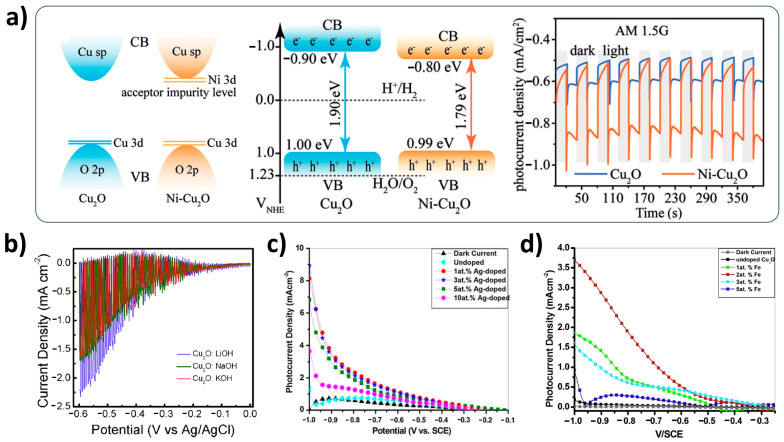
Cation-doped Cu_2_O photocathodes. (**a**) Ni-doped Cu_2_O photocathode fabricated by a one-pot hydrothermal method. Reprinted from [60] with permission from Wiley-VCH Verlag GmbH & Co. KGaA, Weinheim, copyright 2020. (**b**) PEC performances of an alkaline ion (Li^+^, Na^+^, and K^+^)-doped Cu_2_O photocathode. Reprinted from [61] with permission from Hydrogen Energy Publications LLC, Elsevier Ltd., copyright 2018. (**c**) PEC performances of a Ag-doped Cu_2_O photocathode. Reprinted from [62] with permission from Springer Nature, copyright 2013. (**d**) PEC performances of an Fe-doped Cu_2_O photocathode. Reprinted from [63] with permission from Elsevier B.V., copyright 2015.

The poor charge transport characteristics of Cu_2_O should also be improved for developing the efficient Cu_2_O photocathode. It is well-known that cation doping is generally effective to enhance the p-type conductivity of Cu_2_O electrodes, depending on the fabrication method [56,57,58]. Hence, many groups have tried to apply various cations as a dopant to the Cu_2_O photocathode. The Ni-doped Cu_2_O photocathode is representative of these efforts [59,60]. As illustrated in Figure 6a, in the Ni-doped Cu_2_O photocathode, the band gap became narrow due to an acceptor impurity from Ni dopants compared to the pristine Cu_2_O photocathode [60]. It triggered not only accelerated charge separation but also extended light absorption. Furthermore, Ni dopants did not induce the structural distortion of the Cu_2_O photocathode because the ionic radius of Ni^2+^ (0.72 Å) is similar to that of Cu^+^ (0.77 Å). It promotes charge transport, thereby improving PEC performance. Alkaline ions (Li^+^, Na^+^, and K^+^) were also used as a cation dopant by Chen et al. [61]. Although these were all effective to improve charge transport, Li dopants were the most effective to improve the PEC performance (Figure 6b) because lithium(Li)-doped Cu_2_O photocathodes had less defects compared to Na-doped and K-doped Cu_2_O photocathodes. It is mainly due to the different ionic radii of alkaline ions: that of Li^+^ (0.76 Å) is the closest to that of Cu^+^ compared to others (Na^+^: 1.02 Å, and K^+^: 1.38 Å). Silver(Ag)-doped and iron(Fe)-doped Cu_2_O photocathodes were also introduced by Upadhyay et al. [62,63]. They showed the improved PEC performances, compared to those of undoped Cu_2_O photocathodes (Figure 6c,d). It was mainly due to the enhanced charge transport and conductivity by the doping effect. However, the excessive cation doping had negative influences on the PEC performance because it caused a non-homogeneous Cu_2_O photocathode with many defect sites. Therefore, it is important to find the optimal doping level for the improved PEC performance of Cu_2_O photocathodes.

**Figure 7:**
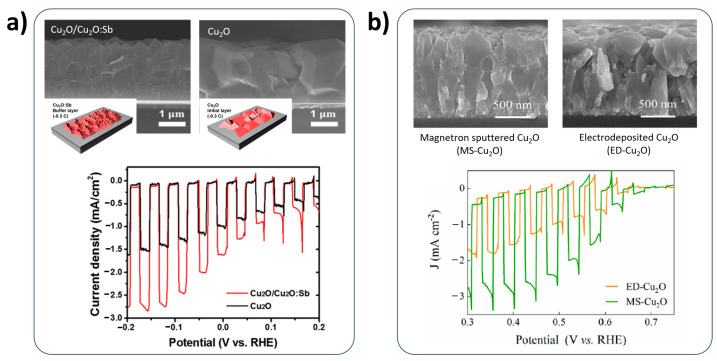
Grain-boundary-controlled Cu_2_O photocathodes. (**a**) Highly-oriented Cu_2_O photocathode with antimony (Sb) incorporated the Cu_2_O (Cu_2_O:Sb) seed layer with its PEC performance. Reprinted with permission from [64]. Copyright 2017 American Chemical Society. (**b**) Comparison of electrodeposited Cu_2_O (ED-Cu_2_O) photocathodes and magnetron-sputtered Cu_2_O (MS-Cu_2_O) photocathodes with their PEC performances. Reprinted with permission from [65]. Copyright 2022 American Chemical Society.

The grain boundary of Cu_2_O photocathodes also affects the charge-transport characteristics. It is advantageous for the efficient Cu_2_O photocathode to alleviate the grain boundaries in the Cu_2_O because they act as a recombination center of charge, reducing the PEC performance. It is controllable to modify the fabrication process. Baek et al. introduced a thin antimony (Sb)-incorporated Cu_2_O (Cu_2_O:Sb) seed layer to grow the highly-oriented Cu_2_O photocathode using electrodeposition [64]. The Cu_2_O:Sb seed layer was synthesized by electrodeposition in the Sb-ions-added copper sulfate aqueous solution. The Sb ions retard lateral diffusion of Cu ions in the solution, resulting in the vertically-oriented Cu_2_O seed layer. It facilitates the growth of highly-oriented Cu_2_O photocathode with less grain boundaries during the electrodeposition process (Figure 7a). Qin et al. recently compared the characteristics of electrodeposited Cu_2_O photocathodes and magnetron-sputtered Cu_2_O photocathodes [65]. They demonstrated that the magnetron sputtering is more feasible to fabricate Cu_2_O photocathodes with less grain boundaries than electrodeposition (Figure 7b). These works reported the improved PEC performance of a Cu_2_O photocathode by the mitigation of grain boundaries in the Cu_2_O photocathode, as shown in Figure 7.

### 2.2. Back Contact Layer

**Figure 8:**
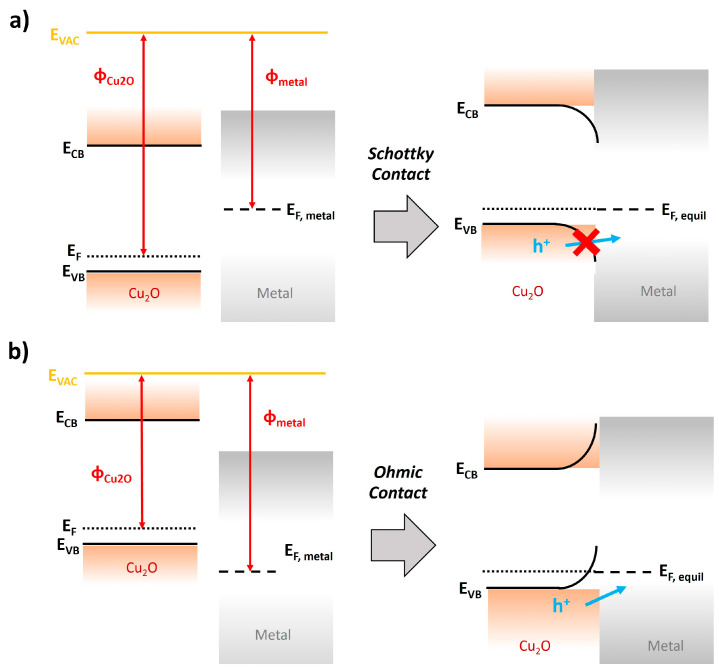
Energy level of the contact interface between Cu_2_O and metal. (**a**) Schottky contact (φ_Cu2O_ > φ_metal_) and (**b**) Ohmic contact (φ_Cu2O_ < φ_metal_) for hole migrations.

The back contact layer is responsible for forwarding generated holes in the Cu_2_O layer to the external load via the conductive substrate. In general, the thin metal film is employed as a back contact layer; thus, the Cu_2_O/metal interface is critical to promote the migration of holes. The characteristic of the Cu_2_O/metal interface is entirely dependent on the work function of Cu_2_O (φ_Cu2O_) and metal (φ_metal_). When the φ_metal_ is smaller than φ_Cu2O_, Schottky contact is formed by the Fermi level equilibration, thereby preventing the hole transportation into the conductive substrate (Figure 8a). On the other hand, the φ_metal_ is larger than φ_Cu2O_, Ohmic contact is formed by the Fermi level equilibration, facilitating the accelerated migration of holes to the conductive substrate (Figure 8b). Hence, the metal should have a larger work function than that of Cu_2_O (4.84 eV) [66] for fitting into the back contact layer for the Cu_2_O photocathode. Table 1 shows metals with a large work function. These are available as a back contact layer for Cu_2_O photocathodes because their work functions are larger than that of Cu_2_O, leading to the Ohmic contact for hole migrations.

A Au back contact layer has been frequently used for Cu_2_O photocathodes [17,32,33,70]. The Cu_2_O photocathode with a Au back contact layer shows a reliable and outstanding PEC performance, due to its excellent hole collection characteristic. Nevertheless, it is disadvantageous for the large-scale application and tandem configuration with a short-band-gap semiconductor, because it is expensive and opaque, respectively. To solve this limitation, Dias et al. developed transparent Cu_2_O photocathodes with a thin Au back contact layer [17]. They found that a thin Au layer below 5 nm is sufficient for collecting holes, similar to a thick Au layer. It also enables the usage of Au to be reduced. The Cu back contact layer is a promising candidate for replacing the expensive Au back contact layer because it is earth-abundant and low-cost [52,71]. Cu_2_O photocathodes with a Cu back contact layer generally showed similar PEC performances with Cu_2_O photocathodes with a Au back contact layer.

**Figure 9:**
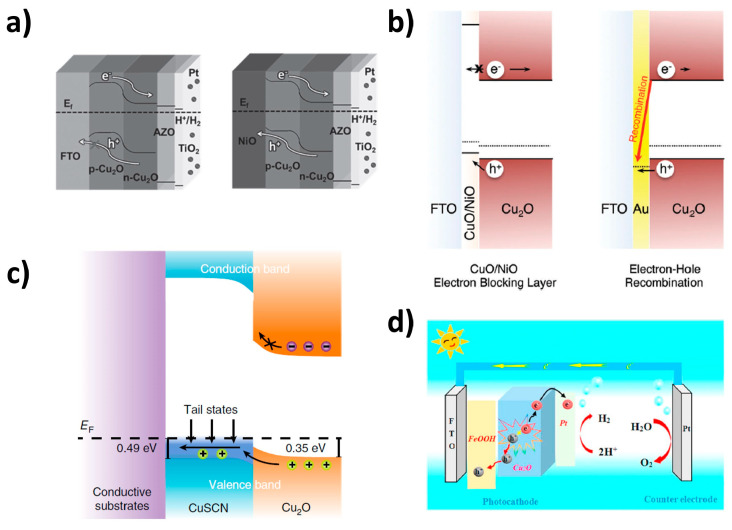
Energy band diagrams of Cu_2_O photocathodes with the non-metallic back contact layer. (**a**) Cu_2_O photocathodes-based FTO substrate and NiO back contact layer. Reprinted from [75] with permission from Wiley-VCH Verlag GmbH & Co. KGaA, Weinheim, copyright 2017. (**b**) Cu_2_O photocathodes with a CuO/NiO hole selective layer and Au back contact layer. Reprinted from [76] with permission from the Royal Society of Chemistry, copyright 2017. (**c**) Cu_2_O photocathodes with a solution-processed CuSCN back contact layer. Reprinted from [77] with permission from Pan et al., copyright 2020. (**d**) Cu_2_O photocathodes with an electrodeposited FeOOH hole transfer layer. Reprinted from [78] with permission from Elsevier B.V., copyright 2020.

Non-metallic materials have been explored as an alternative to the metallic back contact layer for Cu_2_O photocathodes. The main goal for these efforts is to develop the affordable Cu_2_O photocathode avoiding the usage of precious components. Nickel oxide (NiO) is a well-known material as a hole selective layer in perovskite solar cells [72,73,74]. It is also possible to utilize the back contact layer of Cu_2_O photocathodes, because it has a larger work function (5.0 eV) than that of Cu_2_O. As shown in Figure 9a, it forms the Ohmic contact with Cu_2_O for smoothly migrating holes, while the fluorine-doped tin oxide (FTO) substrate forms the Schottky contact with Cu_2_O for interrupting the hole migration due to a shallow band barrier [75]. Son et al. reported the copper nickel mixed oxide (CuO/NiO) hole selective layer for the Cu_2_O photocathode [76]. They fabricated the CuO/NiO thin layer by a sequential metallic Cu/Ni sputtering and annealing process. It efficiently blocks the charge recombination at the interface between the Cu_2_O layer and the conductive substrates due to its huge energy barrier, resulting in an improved PEC performance (Figure 9b). Furthermore, it is quite transparent, which is beneficial for using the Cu_2_O photocathode as a top absorber in the tandem configuration. Copper thiocyanate (CuSCN) was applied as a back contact layer of a Cu_2_O photocathode in the work by Pan et al. [77]. Although the hole transport from Cu_2_O into CuSCN is difficult due to the VB offset in terms of energy level, solution-processed CuSCN thin film facilitates the smooth hole transport from Cu_2_O into conductive substrates by the band-tail states existence, as illustrated in Figure 9c. Moreover, the huge barrier generated by the large CB band offset effectively prevents the charge recombination at the back contact interface. Zhou et al. suggested iron oxide hydroxide (FeOOH) as a hole transfer layer in the Cu_2_O photocathode [78]. Electrodeposited FeOOH thin film promotes the extraction of holes from Cu_2_O into conductive substrates due to its energy level (Figure 9d). Hence, the Cu_2_O photocathodes with the FeOOH back contact layer showed not only an enhanced PEC performance but also an improved stability. 

### 2.3. Overlayer

**Figure 10:**
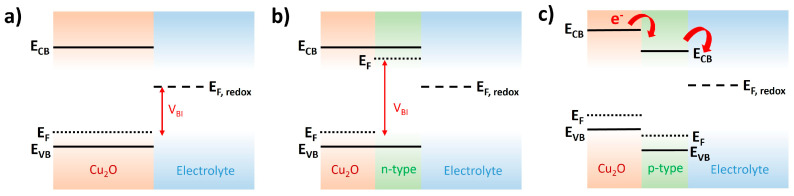
Band diagrams of the Cu_2_O/electrolyte interface. (**a**) Single Cu_2_O photocathode, (**b**) n-type overlayered Cu_2_O photocathode, and (**c**) p-type overlayered Cu_2_O photocathode.

A heterostructured Cu_2_O photocathode with a semiconductor overlayer is a promising strategy to improve the overall water-splitting performance of Cu_2_O photocathodes because the semiconductor overlayer renders the accelerated electrons/holes’ separation and promotes electron transport from Cu_2_O into the water interface. However, the mechanism is slightly different, depending on the intrinsic characteristics of the semiconductor overlayer (n-type or p-type). When the p-n heterostructure is formed with an n-type semiconductor overlayer in the Cu_2_O photocathode, the built-in voltage (V_BI_) created from the difference of E_F_ and E_F,redox_ further increases compared to the single Cu_2_O photocathode (Figure 10a,b) [79]. It promotes the electrons/holes separation and transport. On the other hand, the staircase-type energy levels are created, when the p-p heterostructure is formed with p-type semiconductor overlayer, which has a relatively lower CB and VB than Cu_2_O (Figure 10c) [80]. It accelerates the electron transport into the water interface. Therefore, the n-type semiconductor-overlayered Cu_2_O photocathodes show a remarkable early onset potential, as well as an improved photocurrent density, whereas the p-type semiconductor-overlayered Cu_2_O photocathodes primarily show an improved photocurrent density.

Table 2 summarizes the onset potential and photocurrent density at the HER potential of heterostructured Cu_2_O photocathodes with n-type or p-type semiconductor overlayers. In the case of p-n heterostructures, Cu_2_O photocathodes with a TiO_2_ overlayer show an improved onset potential (earlier onset potential) compared to the single Cu_2_O photocathode. The Cu_2_O/TiO_2_ heterojunction reinforces the band bending, resulting in an improved PEC performance [81,82]. However, TiO_2_ has been widely applied to the protection layer of Cu_2_O photocathodes, rather than the overlayer for the heterojunction effect, because it is an intrinsically stable oxide in the aqueous solution. Although the ZnO overlayer is also effective to improve the charge transport by the formation of heterojunction with Cu_2_O, the aluminum-doped zinc oxide (AZO) overlayer is more efficient for the heterojunction effect because it is more conductive compared to the ZnO overlayer [83,84]. Minami et al. reported that the gallium oxide (Ga_2_O_3_)/Cu_2_O heterostructure improves the photovoltage of Cu_2_O-based solar cells due to the decreased defect levels at the interfaces [85]. Inspired by this work, Li et al. introduced the Ga_2_O_3_ overlayer in the Cu_2_O photocathode for improving the PEC performance [86]. The improved photovoltage by the Ga_2_O_3_/Cu_2_O heterostructure leads to a remarkable enhanced onset potential in the Cu_2_O photocathodes. Pan et al. further improved the PEC performance of Ga_2_O_3_ overlayered Cu_2_O photocathodes with the Cu_2_O nanowire and ruthenium oxide (RuO_x_) HER catalysts [87]. Their devices also showed the improved onset potential, approximately 1.0 V versus RHE. It is advantageous for improving the PEC performance of an unbiased water-splitting system with the Cu_2_O photocathode and photoanode/photovoltaic [88]. Consequently, they succeeded in demonstrating the unbiased all-oxide solar water-splitting system using a Ga_2_O_3_ heterostructured Cu_2_O photocathode and transparent BiVO_4_ photoanode with an operating current density of 2.5 mA cm^−1^, corresponding to an STH efficiency of 3%.

On the contrary to the n-type semiconductor overlayers, most researchers have used a cupric oxide (CuO) overlayer to form the p-p heterostructure for the Cu_2_O photocathode because it is easily prepared using a pure Cu_2_O photocathode via a thermal oxidation process [89,90,91,92]. The electrons are easily moved to the water interface by the staircase-type energy level due to the energy levels of CuO and Cu_2_O, as illustrated in Figure 10c. In addition, the light utilization is also enhanced in the CuO/Cu_2_O heterostructured photocathode, due to a narrow band gap of CuO (1.3~1.7 eV) [93,94]. Hence, the improvement of photocurrent density is prominent, rather than the enhancement of onset potential, in the CuO-overlayered Cu_2_O photocathode. The optimization of the CuO/Cu_2_O heterostructured photocathode has continuously been explored by several groups. Du et al. derived the optimal annealing temperature (650 °C) for fabricating the highly efficient CuO/Cu_2_O photocathode [90]. Jeong et al. found the optimal thickness of CuO for promoting the heterojunction effect [92]. The CuO overlayer with a thickness of approximately 90 nm is optimal for improving the performance of the Cu_2_O photocathode by the CuO/Cu_2_O heterostructure, because it is close to the minority carrier diffusion length of CuO.

### 2.4. Protection Layer

**Figure 11:**
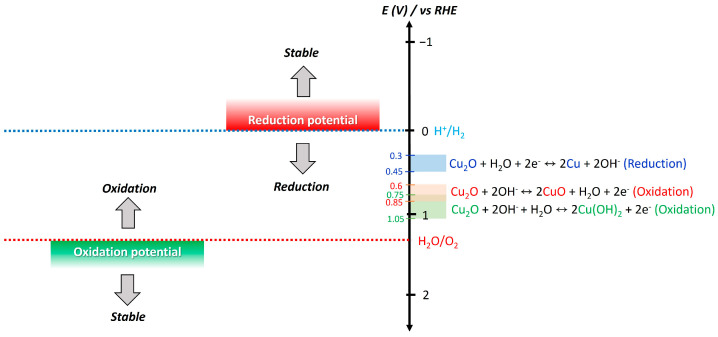
Stability change in the semiconductor in the water and redox potentials of Cu_2_O. It is drawn based on the information in the reported literature [95,96].

The main challenge of the Cu_2_O photocathode is its poor stability against water. It is easily degraded in the aqueous solution within a few minutes, thereby losing its PEC characteristics [32]. Figure 11 illustrates the stability change in the semiconductor in the water. In general, the semiconductor is readily oxidized in the water when its oxidation potential is smaller than the OER potential, while it is simply reduced in the water when its reduction potential is larger than the HER potential, instead of aiding in water-splitting reactions [95]. Cu_2_O meets these two conditions: It is reduced into the metallic Cu in the potential window of 0.3~0.4 V versus RHE, whereas it is oxidized into CuO or copper hydroxide (Cu(OH)_2_) in the potential window of 0.6~1.05 V versus RHE (Figure 11) [96]. Hence, it is extremely unstable in water. This is a reason why the protection layer is an essential component in the Cu_2_O photocathode for durable PEC water splitting.

**Figure 12:**
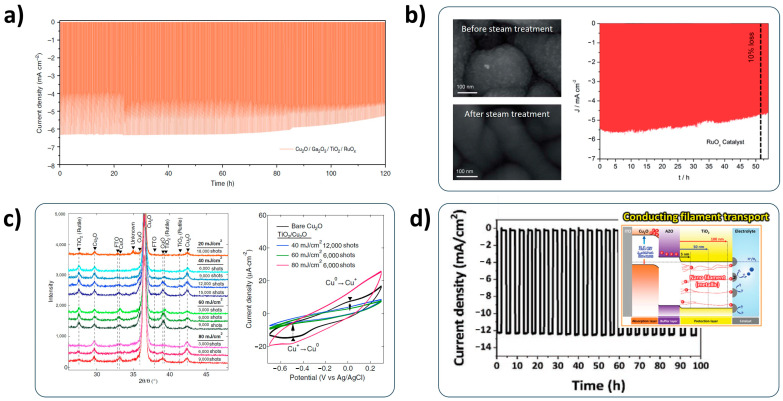
Efforts for improving the stability of Cu_2_O photocathodes using a TiO_2_ protection layer and its modification. (**a**) Long-term stability of Cu_2_O nanowire photocathodes with a Ga_2_O_3_ overlayer and RuOx HER catalysts. Reprinted from [87] with permission from Macmillan Publisher Ltd., Springer Nature, copyright 2018. (**b**) TiO_2_ surfaces before/after steam treatment and the durability of TiO_2_-protected Cu_2_O photocathodes assisted with low-temperature steam treatment. Reprinted from [97] with permission from the Royal Society of Chemistry, copyright 2014. (**c**) Crystallization of the TiO_2_ protection layer induced by laser irradiation and its effect on the stability of Cu_2_O photocathodes. Reprinted from [99] with permission from Elsevier B.V., copyright 2015. (**d**) Stability of Cu_2_O photocathodes with the modified TiO_2_ protection layer using a metallic nano filament design. Reprinted from [100] with permission from Wiley-VCH GmbH, copyright 2021.

Many researchers have used a TiO_2_ thin film as a protection layer for the Cu_2_O photocathode because it is intrinsically stable in water. Its reduction potential is larger than the HER potential; thus, it is robust to corrosion in the aqueous solution. In addition, its CB is beneficial for transporting electrons into the water interface [95]. The TiO_2_ protection layer is generally deposited on the Cu_2_O photocathodes by atomic layer deposition (ALD), which is favorable to deposit the homogeneous thin layer with the thickness of nanometer scale. The benchmark stability of Cu_2_O photocathodes was recorded by Prof. Grätzel’s group using an ALD-deposited amorphous TiO_2_ protection layer in 2018 [87]. Their devices showed a remarkable stability beyond 100 h, as shown in Figure 12a. However, it was gradually degraded after PEC operation for 100 h. This means that the amorphous TiO_2_ protection layer is not sufficient for completely protecting the Cu_2_O photocathode. 

Many efforts have been made to improve its protection capability. Azevedo et al. carried out low-temperature steam treatment on the amorphous TiO_2_-protected Cu_2_O photocathode [97]. Although the crystallinity of TiO_2_ was not changed, the surface of the TiO_2_ became much smoother after the steam treatment (Figure 12b). It cured any defects and cracks in the TiO_2_ protection layer, resulting in an excellent durability with 10% of PEC performance loss over more than 50 h. The crystallization of the amorphous TiO_2_ protection layer is also one of these efforts because the crystalline TiO_2_ is more robust than the amorphous one [96,98]. Nishikawa et al. succeeded in applying the crystalline TiO_2_ protection layer on the Cu_2_O photocathodes using the solution process assisted with excimer laser irradiation [99]. As shown in Figure 12c, the mild laser irradiation with continuous shots induced the crystallization of TiO_2_, especially the rutile phase. It was demonstrated that it efficiently prevents the redox reaction of Cu_2_O, facilitating the stability enhancement of Cu_2_O photocathodes. Furthermore, the structural modification of the TiO_2_ protective layer enables the long-term stability of the Cu_2_O photocathode to improve. A thick TiO_2_ protection layer above 100 nm is beneficial to improve the stability, but it is prone to show a decreased PEC performance due to the disturbance of electron transport. Kim et al. solved this mismatch by introducing a metallic nano filament to reinforce the electron transport in the thick TiO_2_ protection layer [100]. Consequently, the Cu_2_O photocathode showed an excellent stability with a considerable PEC performance for 100 h (Figure 12d). 

A conductive layer also assists to enhance the long-term stability of Cu_2_O photocathodes via the fast electron transfer from the Cu_2_O into the water interface. In this regard, some groups used the metallic layer, such as Au and Ag, for protecting the degradation of Cu_2_O photocathodes [101,102]. Its thickness should be considered because it can block the light irradiation to the Cu_2_O because of its opacity, leading to the decreased PEC performance. A thin metallic layer with tens of nanometer is advantageous to improve the stability without decreasing the PEC performance. Carbon-based material has been explored as a conductive protection layer for Cu_2_O photocathodes. Kunturu et al. developed CuO heterostructured Cu_2_O photocathodes with a 15 nm carbon layer [103]. The thin carbon layer suppresses the photocorrosion of Cu_2_O photocathodes by facilitating fast electron transfer to the surface. Das et al. tried to use a graphene layer, which is more conductive than pure carbon, as a protection layer [104]. They deposited the graphene protective layer on the Cu_2_O photocathode by the chemical vapor deposition (CVD) method. The CVD-fabricated graphene layer had several microcracks, resulting in the degradation of Cu_2_O. They solved this shortcoming by introducing a thin TiO_2_ layer below 10 nm. It patches the microcrack in the graphene layer, thereby efficiently inhibiting the photocorrosion of Cu_2_O. Titanium nitride (TiN) was also used as a protection layer in the work by Diao et al., because it is highly conductive and corrosion-resistant [105]. They controlled the thickness of TiN by adjusting the cycle of ALD deposition to trade off the PEC performance and the stability. Finally, the Cu_2_O photocathodes with the ultra-thin TiN protection layer (8 nm) showed 100% stability during the PEC operation for 1 hr, without critical deterioration of the PEC performance.

Recently, organic materials have also received a lot of attention as a protective layer for Cu_2_O photocathodes. Li et al. introduced the compact polymer layer for protecting Cu_2_O photocathodes [106]. Polyurethane acrylate (PUA) thin film was used to cover the Cu_2_O by a solution process using a viscous urethane acrylate monomer. Their device showed a 98% photo-durability during PEC operation under continuous light illumination for 6 h. Zhang et al. developed phenylethynyl copper (Ph-C≡C-Cu)-protected Cu_2_O photocathodes [107]. The Ph-C≡C-Cu layer was a self-assembled monolayer fabricated by a photothermal method. Hence, the conformal Ph-C≡C-Cu protection layer was successfully applied to the Cu_2_O photocathode. As a result, they constructed a stable unbiased water-splitting system operating for 5 h using the Ph-C≡C-Cu-protected Cu_2_O photocathode with the NiOOH/FeOOH-layered BiVO_4_ photoanode. These protection layers based on organic materials have hydrophobic characteristics, which can inhibit the corrosion of Cu_2_O by avoiding contact with the aqueous solution. Hence, it is thoroughly effective to prolong the stability of Cu_2_O photocathodes. 

### 2.5. Co-Catalysts

In general, HER catalysts have been employed as a co-catalyst on the surface of the protection layer of Cu_2_O photocathodes to reduce the overpotential of the water reduction reaction, leading to the improved PEC performance of Cu_2_O photocathodes. It is well-known that platinum (Pt) is the most promising HER catalyst because of its highly intrinsic activity in the water reduction reaction and low hydrogen adsorption energy [108,109]. Thus, it has been actively adopted for improving the PEC performance of Cu_2_O photocathodes [32,43,110,111,112,113]. Nevertheless, it is unfavorable for long-term stability because it detaches from the Cu_2_O photocathodes during PEC operation. It is mainly attributed to the weak bonding of the Pt and TiO_2_ protection layer [96]. RuO_x_, which was suggested by Tilley et al. [114], is an alternative HER catalyst to Pt for the durable PEC operation of Cu_2_O photocathodes. It is more robust than Pt due to the strong bonding with the TiO_2_ protection layer. Hence, the RuO_x_-photoelectrodeposited Cu_2_O photocathode showed a better stability with a competitive PEC performance, compared to the Pt-catalyzed Cu_2_O photocathode. However, these components (Pt and RuO_x_) are not suitable for the economical PEC water-splitting system because they are a noble metal. Therefore, many researchers have pursued the application of inexpensive HER catalysts with low-cost materials to the Cu_2_O photocathodes for obtaining the economic feasibility of the PEC water-splitting system.

**Figure 13:**
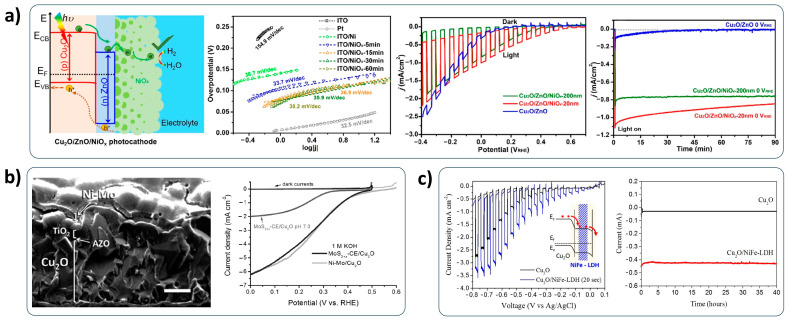
Ni-based HER catalyst-decorated Cu_2_O photocathodes. (**a**) Schematic diagram of NiO_x_ decorated ZnO/Cu_2_O photocathodes, HER catalytic activity of NiO_x_ composite, PEC performance and stability of NiO_x_-decorated ZnO/Cu_2_O photocathodes. Reprinted with permission from [116]. Copyright 2020 American Chemical Society. (**b**) Morphology and PEC performance of Ni-Mo-decorated Cu_2_O photocathodes with a TiO_2_ protection layer. Reprinted from [117] with permission from Wiley-VCH Verlag GmbH & Co. KGaA, copyright 2015. (**c**) PEC performance and stability of Ni-Fe LDH-decorated Cu_2_O photocathodes and bare Cu_2_O photocathodes. Reprinted from [118] with permission from Qi et al., copyright 2016.

Ni-based materials are a promising candidate as a low-cost HER catalyst. Lin et al. deposited the NiO/nickel hydroxide (Ni(OH)_2_) composite on the Cu_2_O nanowire photocathode by the solution process and sequential annealing process [115]. They found that the NiO/Ni(OH)_2_ composites allow for an improved charge transfer by retarding charge recombination via the synergy effect of NiO and Ni(OH)_2_. Hence, the NiO/Ni(OH)_2_ composite-decorated Cu_2_O nanowire photocathode showed an enhanced PEC performance. Recently, Jian et al. developed a highly efficient NiO_x_ composite (mixture of Ni and NiO) HER catalyst fabricated by vacuum evaporation deposition and heat treatment [116]. As shown in Figure 13a, it showed a remarkable HER activity (Tafel slope of 35.9 mV dec^−1^), which was very close to the HER activity of Pt (Tafel slope of 32.5 mV dec^−1^). Thus, the NiO_x_-catalyzed Cu_2_O photocathode showed a positive shift of onset potential from 0.2 V to 0.6 V versus RHE. Furthermore, it showed quite a stable PEC performance for 90 min. This means that the NiO_x_ composite plays a role as not only an HER catalyst but also as a protection layer for Cu_2_O photocathodes. The electrodeposited Ni-Mo (Molybdenum) alloy, which was suggested in the work by Morales-Guio et al. [117], also significantly reduced the HER overpotential in an alkaline aqueous solution. The Ni-Mo-decorated Cu_2_O photocathode produced a high photocurrent density of −6.3 mA cm^−2^ at 0 V versus RHE without any dark currents in the basic solution (Figure 13b). Qi et al. successfully applied Ni-Fe (Iron)-layered double hydroxide (LDH) to the Cu_2_O photocathode [118]. The electrodeposited Ni-Fe LDH catalysts boosted the electron transfer into the water interface by the appropriate band alignment with Cu_2_O. Consequently, the Ni-Fe LDH co-catalyzed Cu_2_O photocathode showed a seven-fold increase in PEC performance with remarkable stability for 40 h compared to a bare Cu_2_O photocathode (Figure 13c).

**Figure 14:**
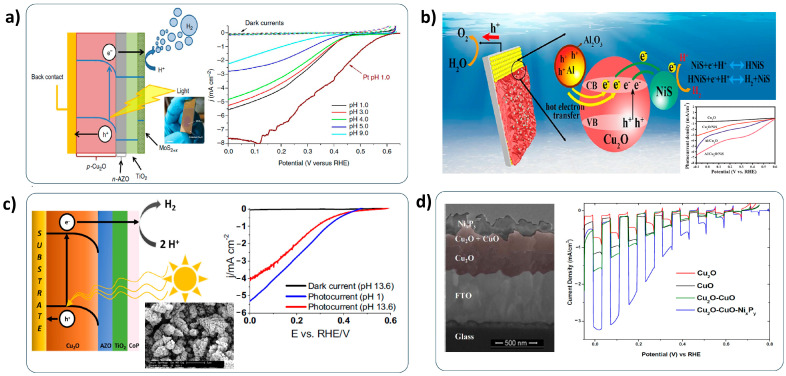
Transition metal sulfides- and phosphides-catalyzed Cu_2_O photocathodes. (**a**) Schematic diagram and PEC performance of MoS_x_-decorated Cu_2_O photocathodes. Reprinted from [119] with permission from Springer Nature Limited, copyright 2014. (**b**) Schematic diagram and PEC performance of Al-NiS-catalyzed Cu_2_O photocathodes. Reprinted from [120] with permission from Elsevier B.V., copyright 2019. (**c**) Schematic diagram and PEC performance of CoP-decorated Cu_2_O photocathodes. Reprinted from [121] with permission from Elsevier Ltd., copyright 2017. (**d**) Morphology and PEC performance of a NiP-deposited CuO/Cu_2_O photocathode. Reprinted from [122] with permission from Royal Society of Chemistry, copyright 2018.

Transition metal sulfides have been considered as a good HER catalyst for Cu_2_O photocathodes, due to their high conductivities. Morales-Guio et al. deposited molybdenum sulfide (MoS_x_) co-catalysts on the TiO_2_-protected Cu_2_O photocathode by photoelectrodeposition [119]. The MoS_x_-loaded Cu_2_O photocathode showed an outstanding PEC performance with a photocurrent density of −5.7 mA cm^−2^ at the HER potential, corresponding to an STH efficiency of 7%, in the strong acidic electrolyte (pH 1), as shown in Figure 14a. Although the condition of the electrolyte was extremely harsh, their devices survived with a reliable PEC performance for 10 h. Chen et al. developed nickel sulfide (NiS)-combining aluminum (Al) nanoparticles HER catalysts for the Cu_2_O photocathode [120]. NiS HER catalysts were deposited by a successive ionic layer adsorption reaction (SILAR) method. They demonstrated that the NiS attracts more protons (H^+^) into the surface of Cu_2_O photocathodes and forms enriched electron conditions by the Ni-H bonds, resulting in a reduced HER overpotential. As a result, the Al nanoparticles NiS-co-catalyzed Cu_2_O photocathode showed an improved PEC performance, along with the plasmonic effect by the Al nanoparticles (Figure 14b).

Transition metal phosphides have also been explored as low-cost HER catalysts for Cu_2_O photocathodes because they have an excellent catalytic activity for HER. Cobalt phosphide (CoP) catalysts were successfully adopted for the Cu_2_O photocathode in the work by Stern et al. [121]. Nanoflower-like CoP catalysts showed an extremely low HER overpotential of approximately 97 mV in a strong acidic condition to reach a current density of 10 mA cm^−2^. Consequently, their CoP-decorated Cu_2_O photocathodes showed a considerable photocurrent density of −5.3 mA cm^−2^ at the HER potential in the strong acidic electrolyte, which is a favorable condition for the water reduction reaction (Figure 14c). Chhetri et al. developed nickel phosphide (NiP)-decorated Cu_2_O photocathodes [122]. They successfully deposited NiP catalysts on the CuO heterostructured Cu_2_O photocathode by the pulse plating electrodeposition. Their devices showed a noticeably improved PEC performance by the fast electron transfer into the water interface due to the NiP catalysts compared to the bare Cu_2_O photocathodes (Figure 14d).

## 3. Outlook and Future Research Directions

Four aspects should be considered for the practical PEC water-splitting system: efficiency, stability, cost, and mass production. In terms of efficiency and cost, Cu_2_O is a frontrunner photocathode material for the practical PEC water-splitting system, because the state-of-the-art Cu_2_O photocathode shows a high STH efficiency above 10%, which is a benchmark for the commercialization. In addition, it is an earth-abundant material. However, it is necessary to further improve its stability above 100 h for the durable PEC water-splitting operation. Furthermore, upscaling is essential for the mass production of hydrogen via the PEC water-splitting system based on Cu_2_O photocathodes. Therefore, future research on the Cu_2_O photocathode should move forward based on strategies as below.

1. Cu_2_O absorber: The further improvement of electron transport capability will be necessary. To this end, doping is the most efficient strategy. In the case of this strategy, the ionic radius of the dopant should be similar to that of Cu^+^ ions for reducing the defects on the Cu_2_O film. In addition, the doping level should be optimized for improving the electron transport in the Cu_2_O photocathodes, because the excessive doping has a negative influence on the PEC performance. The fabrication of a high-quality Cu_2_O film with less defects or grain boundaries is also advantageous to improve the electron transport in the Cu_2_O photocathode. Furthermore, the development of transparent Cu_2_O photocathodes with efficient PEC performance paves the way for developing the efficient PEC-PEC or PEC-PV water-splitting system with short-band-gap materials;
2. Back contact layer: The development of an alternative back contact layer to the expensive Au back contact layer is a main goal of this component. In the case of metal, its work function should be higher than that of Cu_2_O. Moreover, control of the opacity is necessary for the development of transparent Cu_2_O photocathodes. A semiconductor with a huge energy barrier is a good option because it efficiently hinders the electron recombination at the interface. In this case, the suitable deposition method of Cu_2_O should be considered on the semiconductor-based back contact layer;
3. Overlayer: In the case of n-type overlayers, the created photovoltage in contact with Cu_2_O should be considered because it motivates the charge separation in the p-n junction with Cu_2_O. In the case of p-type overlayers, the exploration on the alternative material to the CuO overlayer with the proper energy level for enhancing the electron transfer into the water interface is a good strategy for the future research direction;
4. Protection layer: Although the amorphous TiO_2_ protection layer is highly efficient for protecting a Cu_2_O photocathode against the corrosion, it is still not sufficient due to its pinholes or defects. Hence, the reduction in pinholes or defect of the amorphous TiO_2_ protection layer is useful for further improvement of its protection capability. The crystallization method of the TiO_2_ protection layer without damaging the Cu_2_O photocathode is also feasible to improve the stability of the Cu_2_O photocathode without a decreased PEC performance. The technique to form the hydrophobic surface on the Cu_2_O photocathode is a promising strategy to improve the stability of Cu_2_O photocathodes;
5. Co-catalysts: The development of non-noble HER catalysts and the alleviation of noble components in HER catalysts is essential for the low-cost PEC water-splitting system. Although various HER catalysts have recently been developed [123], the deposition method should be considered for successfully applying to the Cu_2_O photocathode. Furthermore, the bonding of the HER catalyst with a Cu_2_O photocathode should be concerned for the durable Cu_2_O photocathode because it is directly related to the stability;
6. Upscaling: The reported high PEC performance of Cu_2_O photocathodes is normally based on a small scale below 1 cm^2^. In general, it is significantly reduced in the large-scale Cu_2_O photocathodes [124]. Therefore, the research on maintaining its high PEC performance in the large-scale Cu_2_O photocathodes is necessary, such as a novel design. Although a few groups have recently reported their works on the large-scale Cu_2_O photocathode [124,125], more vigorous efforts on this are still essential for realizing the mass production of hydrogen via the PEC water-splitting system based on the Cu_2_O photocathode in the future.

## 4. Conclusions

Cu_2_O photocathodes have rapidly advanced for the practical PEC water-splitting system. Although some challenges remain to be overcome, such as the stability and the upscaling, the commercialization of the PEC water-splitting system using Cu_2_O photocathodes is remarkably optimistic in the future, due to its potential for a high PEC performance and economic feasibility. Key strategies on each component in the Cu_2_O photocathode, as suggested in this review paper, provide a shortcut for realizing this optimistic prospect. Furthermore, it will be the cornerstone of the successful entrance of a practical PEC water-splitting system into the eco-friendly hydrogen-fuel-based economy in the near future.

## Figures and Tables

**Table 1:** Available metals as a back contact layer of the Cu_2_O photocathode and their work functions [66,67,68,69].

Metal	Au	Pt	Cu	Ni	Co	Pd	Ir
Work function (eV)	5.1~5.2	5.35~5.65	5.1	5.15	5.0	5.12~5.30	5.25~5.27

**Table 2:** Onset potential and photocurrent density at the HER potential of heterostructured Cu_2_O photocathodes with n-type or p-type semiconductor overlayers. Some devices consist of a TiO_2_ protection layer and HER catalysts, as well as overlayers.

Overlayer	Device	Onset Potential (V versus RHE)	Photocurrent Density (mA/cm^2^, 0 V versus RHE)	Ref.
TiO_2_	Cu_2_O/TiO_2_	0.0 (versus Ag/AgCl)	−0.8 (−1.0 V versus Ag/AgCl)	[81]
TiO_2_	Cu_2_O	0.37	−0.52	[82]
	Cu_2_O/TiO_2_	0.42	−1.40	
	Cu_2_O/TiO_2_/NiFe	0.50	−2.60	
	Cu_2_O/TiO_2_/rGO/NiFe	0.54	−3.71	
ZnO	Cu_2_O/ZnO/TiO_2_/Pt	0.65	−4.00	[83]
AZO	Cu_2_O/ZnO	0.50	−1.60	[84]
	Cu_2_O/AZO	0.63	−2.90	
Ga_2_O_3_	Cu_2_O	0.50	-	[86]
	Cu_2_O/Ga_2_O_3_/Pt	0.90	−4.00	
	Cu_2_O/Ga_2_O_3_/TiO_2_/Pt	1.00	−6.50	
Ga_2_O_3_	Cu_2_O/AZO/TiO_2_/RuO_x_	0.50	−8.00	[87]
	Cu_2_O/Ga_2_O_3_/TiO_2_/RuO_x_	1.00	−9.60	
CuO	Cu_2_O	0.45	−0.21	[89]
	Cu_2_O/CuO	0.80	−2.47	
CuO	Cu_2_O	0.0 (versus Ag/AgCl)	−0.06 (−0.3 V versus Ag/AgCl)	[90]
	Cu_2_O/CuO	0.1 (versus Ag/AgCl)	−0.26 (−0.3 V versus Ag/AgCl)	
CuO	Cu_2_O/CuO	0.60	−2.70	[91]
CuO	Cu_2_O	0.50	−0.15	[92]
	Cu_2_O/CuO	0.50	−1.20	

## Data Availability

The data presented in this study are available on the request from the corresponding author.
